# Measuring Food Insecurity: The Problem with Semantics

**DOI:** 10.3390/foods12091816

**Published:** 2023-04-27

**Authors:** Rachel M. Sumsion, Haylie M. June, Michael R. Cope

**Affiliations:** Department of Sociology, Brigham Young University, Provo, UT 84602, USA; rm553@byu.edu (R.M.S.); hjune@byu.edu (H.M.J.)

**Keywords:** food insecurity, food security, measurement, hunger, levels of analysis

## Abstract

Rising rates of hunger and food insecurity have sparked a major re-evaluation of all aspects of food systems. Because of the multifaceted nature of food insecurity, however, determining what actions should be taken is challenging, especially since reevaluation efforts are led by experts from several disciplines and there is no consensus about which indicators should be used and how they should be measured. Confusion surrounding the meaning of the terms ‘food security’ and ‘food insecurity’ has contributed to this lack of consensus. As indicators inform action, such confusion has slowed those committed to alleviating hunger in identifying the most pressing targets. This review highlights (1) the importance of clearly defining food security and food insecurity and (2) how such definitions affect measures of food insecurity in the United States. While some might say that definitions are an issue of the past or a trivial matter of semantics, we believe that the world’s present rates of hunger and malnutrition are attributable, at least in part, to the lack of consensus on these definitions and their accompanying measurements and indicators. Although the present review can be helpful to academics and policy makers, the primary purpose is to be a resource to those involved in the day-to-day production of food, such as ranchers and farmers by providing an overview of definitions, indicators, and measurements used when discussing food security.

## 1. Introduction

The right to food has been recognized as an international human right for almost 75 years [1]. Since the 1950s, dozens of organizations have worked to alleviate hunger and improve food systems and access to food [2]. However, despite these efforts, progress in combatting hunger and food insecurity has stalled and, in some cases, even been reversed in recent years due in part to the COVID-19 pandemic and Russian–Ukrainian conflict. Indeed, “after remaining relatively unchanged since 2015… the number of people affected by hunger since (2019 grew) by about 150 million” [3]. In 2020, an estimated 828 million people went hungry [3], prompting researchers and policymakers to re-evaluate global food systems [4]. Unfortunately, progress in such efforts has been slowed, however, due to various understandings of the terms ‘food security’ and ‘food insecurity’ as well as their accompanying indicators. 

Research on food insecurity spans a variety of disciplines. Agriculturists, anthropologists, economists, nutritionists, sociologists, and others have all made valuable contributions on the subject [5]. The value of these multidisciplinary efforts cannot be understated; however, significant variation exists between each discipline’s definitions and methods of measuring food security. Despite international consensus supposedly being reached in 1996 on the definition of food security (the 1996 World Food Summit definition of food security (updated in 2009) is: “Food security exists when all people, at all times, have physical, social and economic access to sufficient, safe and nutritious food to meet their dietary needs and food preferences for an active and healthy life” [2]), many definitions of ‘food security’ and ‘food insecurity’ are still commonly used and propagated today between and within disciplines. As definitions precede measurements, measurements produce indicators, and indicators inform action, this is problematic [6]. Comprehensive, valid measurements of food security are needed to ensure accuracy in current and future projections of food insecurity. Such measurements are built upon a foundation of common definitions. This review serves as a call to reestablish consensus in the definitions and measures of food security and insecurity as without agreement on who is experiencing food insecurity, where they are located, when they are most vulnerable, or why they are experiencing it, little can be done to effectively combat it [6]. 

This paper presents an overview of food security/insecurity, by summarizing current and common definitions of the terms, as well as different indicators and measurements of said indicators. It highlights notable similarities and differences between definitions and makes recommendations for a renewed focus to be placed on the use of consistent definitions between and within disciplines. Following this semantic overview, we illustrate how different definitions of food security and insecurity affect measurements and indicators of the topic in the United States. To conclude, we recommend that standardized definitions are reviewed and reemphasized.

Although some experts may say that definitions are an issue of the past or that this topic should be left to semanticists, we believe that the world’s tragic rates of hunger and malnutrition are partially attributable to the confusion surrounding what ‘food insecurity’ is and how to measure it [7]. The current paper contributes to the literature by providing a comprehensive review of the language of ‘food insecurity’ [8]. While the current paper can be useful to academics across disciplines and to policy makers, the primary audience of the paper are members of communities that contribute to food production on a day-to-day basis, such as farmers and ranchers. We believe that providing a comprehensive review for this audience can be used to inform policy, help those in food production receive needed government grants, and ultimately help alleviate food insecurity.

## 2. Review

### 2.1. Why Food Insecurity? 

Since 1948, the right to food has been internationally recognized as a basic human right [9]. Despite a broad consensus about what this means, together with many valiant efforts to combat malnutrition in all its forms, it is estimated that over 820 million people worldwide go hungry and around 2 billion people experience food insecurity each year [4,10,11]. Food insecurity is a serious issue as it is related to a variety of adverse physical, mental, and social health outcomes for both adults and children. Adults who experience food insecurity are more likely to contract noncommunicable diseases (such as diabetes, hypertension, hyperlipidemia, or chronic heart disease), have a reduced fertility rate, a decreased basal metabolic rate, micronutrient deficiencies (particularly protein, Vitamin A, B Vitamins, calcium, iodine, iron, and zinc), food allergies, or be overweight or obese [4,9,12,13,14,15,16,17]. Children experiencing food insecurity have higher rates of cognitive, behavioral, and social problems, and are more likely to be stunted, wasted, have poor oral health, anemia, asthma, or suicidal ideations [8,13,15,18,19,20]. These adverse effects, along with others, have been linked to people experiencing food insecurity spending over USD 1860 more per capita annually on healthcare than people who are food secure [15]. In the most extreme cases, food insecurity can lead to death for both children and adults. 

In response to these grim findings, and in recognition that food security is one of the world’s most pressing issues, government and food security experts have been meeting for years to discuss hunger and food security [10,21]. Their combined efforts have helped to reduce the number of those affected by hunger and malnutrition. In 2016, the United Nations (UN) announced that its second Sustainable Development Goal was to “end hunger, achieve food security and improve nutrition and promote sustainable agriculture” by 2030. To do so, they planned to double small farmers’ productivity and incomes, increase agriculture investment, and ensure food markets’ proper functioning [15,22]. While the UN’s inspiring goal invigorated efforts to improve food systems, and a significantly larger number of studies on food insecurity—including issues of sustainability, the environment, socioeconomics, culture, politics, and governance—have been conducted over the past nine years, rates of worldwide food insecurity have been on the rise since 2014 [4] with recent reports projecting that nearly 670 million people will still be undernourished in 2030 [3].

### 2.2. Defining Food (In)Security

‘Hunger’ is a familiar, but multi-layered term [23]. In academia, scholars often refer to it as the “painful or uneasy sensation caused by a lack of food” [24] (p. 1560) [25]. However, due to its subjective nature, since 2006 there has been no officially recognized metric of ‘hunger’ [26]. Instead, researchers and policymakers measure the food security and food insecurity of nations, regions, households, and individuals—levels of analysis which seldom yield the same results. While ‘hunger’ and ‘food insecurity’ are not synonymous, they are closely related, with hunger being a potential consequence of food insecurity [27,28].

Ironically, while measures of ‘hunger’ have been rejected for their subjectivity, the concepts of ‘food security/insecurity’ are also defined and measured without objectivity [29]. While the terms have been around since the 1950s, they are understood and applied differently according to the context and region in which they occur [30,31]. The term ‘food security’ was first defined at the World Food Conference in 1974 as “(the) availability at all times of adequate world food supplies of basic foodstuffs to sustain a steady expansion of food consumption and to offset fluctuations in production and prices” [32]. As understanding of the causes and consequences of food security/insecurity have shifted and expanded since that time, however, definitions have continuously evolved. Food security/insecurity has been described as a flexible, multidimensional concept, and has been understood and applied differently by sociologists, agriculturists, political scientists, nutritionists, and economists throughout the years [33,34]. In the past especially, various viewpoints contributed to a “bewildering number of paradigms and points of view” in the literature [9] (p. 50). For instance, in the 1990s, Smith, Pointing, and Maxwell said that “there is no single definition (of food security or food insecurity)… but rather a complex weave of inter-related strands which are adjusted to suit the needs and priorities of individual users” [35] (p. 136). To illustrate their point, they compiled an annotated bibliography of close to two hundred definitions of these terms and called for the development of a comprehensive definition [35]. As a result, at the 1996 World Food Summit, a new definition of food security was proposed and ratified [36], to which only minor adjustments have been made since. The most recent agreed upon definition states that:

Food security exists when all people, at all times, have physical, social, and economic access to sufficient, safe, and nutritious food that meets their dietary needs and food preferences for an active and healthy life [36].

While this definition has largely been accepted and is used by most scholars well-versed in the literature, definitions of food security and its counterpart, food insecurity, still vary between disciplines and countries. For instance, while the Life Sciences Research Office’s 1990 definition of food insecurity (food insecurity is the limited or uncertain availability of nutritionally adequate and safe foods, or the limited or uncertain ability to acquire acceptable foods in socially acceptable ways [24]) is often used in the United States, it has not been widely adopted elsewhere.

While Smith et al.’s work helped clarify the concepts of food security and food insecurity in the 1990s, a good number of definitions of the concepts are still used and propagated today in academic as well as grey literature. Inspired by and drawing from the work of Smith et al., Table A1 and Table A2 illustrate the relevance of this issue as each of the 52 definitions presented (19 for food security and 33 for food insecurity) have been cited as primary definitions of the concepts in scholarly literature since 2000 (four years after the UN’s World Food Summit definition was ratified to allow time for adoption). While we acknowledge that the definitions of individual researchers unfamiliar with the semantic history of food insecurity should not be given the same weight as definitions produced through more intensive processes, we maintain that to non-academic audiences and/or those less familiar with food security literature, the continued use of so many definitions can have negative repercussions, especially since much of the nuance between these definitions is far from insignificant when it comes to measuring food insecurity [9,10,35,37].

### 2.3. Varying Definitions of Food Security

If poor data leads to poor policies, then rising rates of food insecurity may be attributable, at least in part, to a lack of consensus in the measures and corresponding indicators of ‘food security’ and ‘food insecurity.’ Definitions, targets, and cut-off points for indicators such as ‘adequate’, ‘sufficient’, ‘nutritious’, ‘acceptable’, or ‘preferred’ foods vary from organization-to-organization, fragmenting the efforts of organizations committed to eradicate hunger, despite reaching a consensus during the World Food Summit in 1996 [7]. The World Food Summit definition has varying dimensions and while it is widely adopted in the literature in the United States, consistent indicators and measurements have not. To better inform policy decisions and develop more effective interventions, clear data, indicators, and measurements are needed. Each of these aspects is dependent upon sound definitions and clarity in regard to which specific dimensions of a definition are being measured [38,39]. 

Without concretely defining the bounty of food security or the threat of food insecurity, actionable solutions to the latter may remain out of reach [40]. We recognize that there is danger in relying on a single definition as a “definition implies a choice, a particular way of seeing a problem among a range of alternatives (and) policy is determined in part by that choice” [41] (p. xii). Accordingly, some scholars plausibly argue that a single, universal, “catch-all” definition does not adequately address the complexities of food security or food insecurity [5,37,39,42,43] (p. 1023–1024). For this reason, multifaceted definitions are needed to generate broad agreement among policymakers and practitioners with diverse emphases. Without such agreement, inconsistencies will continue to exist between various organizations’ measurements, policies, and actions [16]. Care should be taken not to conflate ‘food security’ or ‘food insecurity’ with other terms. For instance, in many cases, a ‘food security’ organization’s focus may be more appropriately described by terms such as ‘nutrition security,’ ‘hidden hunger,’ ‘food insufficiency,’ ‘food access,’ ‘food capacity,’ ‘food resilience,’ ‘food rights,’ or ‘food sovereignty’ [5,44,45]. Additionally, the multi-dimensionality of food security should be recognized when developing and using measurements for it [39]. 

For effective policies to be made, common measurements and agreed upon definitions of ‘food security’ and ‘food insecurity’ are needed [8,38]. Table A1 and Table A2 present many definitions that have been used to describe these concepts. Most broadly accepted definitions recognize that ‘food security’ and ‘food insecurity’ are multifaceted concepts. ‘Four pillars’ are commonly agreed upon [8,46,47]: food availability (supply and production), food access (economic and physical), food utilization (use), and food stability (consistency) [15]. In recent years, calls to expand our conceptual definition of food security has led to the proposal of two additional pillars: agency (empowerment in consumption and production) and sustainability (long-term impacts) [37,48]. Despite such similarities, differences in aspects such as the level of analysis to which the definition applies, what is meant by phrases such as ‘enough food,’ ‘adequate food,’ ‘preferred food,’ or ‘culturally or socially acceptable means,’ and the relative importance or unimportance of self-perceptions impact measurements, rates, and understandings of the severity of food security and insecurity. Such differences are crucial for policymakers to be aware of and understand. We address this point in the following section by exploring how definitional differences can lead to variation in measures of food security/insecurity by implying different levels of analysis and understandings of phrases.

#### 2.3.1. Levels of Analysis

Although hunger is a state that is experienced on the individual level, food security and food insecurity are often conceptualized at various levels of analysis [18,35]. The most common levels of analysis are ‘global,’ ‘national,’ or ‘regional’ (which generally concern the level and reliability of the availability of food supplies), ‘household’ (which generally means the household’s economic, physical, or socio-cultural access to food), or ‘individual’ (often someone’s economic, physical, or socio-cultural access and entitlement to food) [31,49]. Table 1 summarizes each of the levels of analysis.

Before the 1980s, food insecurity was considered almost exclusively at the global or national level and was understood to be an issue of food supply; that is, the main driver of food insecurity was thought to be the lack of food [48]. Consequently, most efforts to address food insecurity focused on improving agricultural yields and regulating national reserves. Over time, however, researchers recognized that the availability of food alone was not sufficient to combat hunger. This fact was widely accepted in the early 1980s with the publication of Amartya Sen’s Poverty and Famines in which he argued that a lack of food entitlement—unequal access to food by production, trade, one’s own labor, or transfers—not a lack of food itself, was a principal driver of food insecurity [50]. From this time forward, “food access” which is measured at the household and individual level has been recognized as a principal component of food security [9,50].

While progress has accompanied this analytical shift, choosing to operationalize food security/insecurity at any given level of analysis requires making assumptions about that level [9]. Consider the ‘household’ level of analysis. What is meant by ‘household’? While it was originally intended to mean individual family units, today some consider any occupants under a single roof, including non-family members such as tenants, boarders, roommates, or other non-dependents as ‘household’ members.

Further, assuring that enough food is allocated at the household level does not necessarily translate to people receiving enough food at the individual level as the distribution of food within households can still be unequal to people’s specific needs [8,50]. For instance, maternal buffering occurs when mothers consume less food to give their children more [1]. Such buffering, along with other similar coping strategies, would pass undetected if organizations solely relied upon household level measures. If organizations relied solely upon individual level measures, however, they could fall victim to overestimating the severity of such a family’s experience. Suffice it to say, in cases when levels of analysis are conflated, rates of food insecurity can be over-or-under-exaggerated [50]. For this reason, deciding what level of analysis the concepts of food security and insecurity are meant to refer to might clarify the mission of organizations and effectively narrow their focus in helping those experiencing food insecurity. Of course, the goal of doing so would not be to ignore other levels of food security or insecurity, rather to clarify those that are most important to organizations. Terms such as ‘food supply’ (global, national, or regional levels of analysis) or ‘food access’ (household or individual levels of analysis) might be profitably used to clarify these differences.

#### 2.3.2. Same Words, Different Ideas

Cultural differences and expectations impact the interpretations of definitional constructs, or the different dimensions of a definition. In measuring food security and insecurity, these different understandings can have a significant impact on food security data. For instance, what is meant by ‘enough’ or ‘sufficient levels’ of food significantly affects reported rates of food insecurity. ‘Enough’ can refer to a variety of indicators such as individual energy requirements (measured as calories or macronutrients (often revised according to age, gender, and activity rates) [8] (p. 1), personal nutrition requirements (measured in micronutrients; specifically, vitamin A, iron, and iodine [9]), or personal perceptions (measured according to self-reported surveys [50]). Several of the variations in how such indicators are used can be explained by the definitions influencing them. Some definitions imply that ‘enough’ means a minimal level of food consumption (see 51–54), others mean the amount of food required to meet nutritional needs (see 18, 24, 55–59), and still others mean the amount necessary to live an “active, healthy life” (see 2, 26, 24, 50, 60–67) (see Table 2).

Similar analyses of definitional constructs such as ‘adequate foods’, ‘preferred foods’, and ‘culturally or socially acceptable means’ are also important to understand (see Table A3, Table A4 and Table A5). Although some scholars have made efforts to clarify these constructs (for instance, Pinstrup-Andersen defines ‘preferred foods’ as “foods that are socially and culturally acceptable and consistent with religious and ethical values” [50] and Frongillo and Horan define ‘socially unacceptable means’ as “buying food on credit, using food pantries, or… asking friends and relatives for money for food” [47] (p. 30), most definitions lack such specification. We believe that each of these constructs needs clarifying to be of value to policymakers. In their present form, they are largely subjective, and their applications vary significantly between contexts [39]. This presents difficulties in developing comparable measurements and coordinated plans for action. By finding common ground for definitions and measurements, a more comprehensive understanding of food security and food insecurity will emerge and lead to meaningful progress in combatting world hunger.

### 2.4. Measuring Food Security

Measures of food security are continually being refined and improved, even though scholars generally agree than food security rests on the four pillars of availability, access, utilization, and stability [37,48,68]. In 2006, Webb et al. identified that many measurements were (1) shifting their focus from measures of availability and utilization to measures of access, (2) shifting from objective to subjective measures, and (3) placing more of an emphasis on direct rather than proxy measures of food security [69]. In 2012, the Committee on World Food Security recommended that all measurements of food insecurity be based on human rights, ensure accountability, involve all relevant stakeholders, be easily understandable, and build upon rather than duplicating national capacities [68]. Recently, some food security definitions have expanded to include agency and sustainability as pillars to understanding the right to food [37,48]. 

Some of the most widely recognized measures of food security in the United States today include Anthropometric measures, Coping Strategies Index (CSI), Domestic Food Price Volatility, Food Consumption Score (FCS), Food Insecurity Experience Scale (FIES), Global Food Security Index (GFSI), Global Hunger Index (GHI), Household Consumption and Expenditure Surveys (HCES), Household Dietary Diversity Score (HDDS), Household Food Insecurity Access Scale (HFIAS), Household Pulse Survey (HPS), Months of Adequate Food Provisioning (MAHFP), Prevalence of Undernourished (PoU), and the Relative Dietary Supply Index (see Table 3). Each of these indicators assess slightly different aspects of food security. For instance, while some of these indicators focus on macro-measures at the global, national, or regional levels of analysis, others focus on micro-measures at the household and individual levels. Consequently, different measures prioritize selective pillars of food security as food supply (availability) is typically measured on a macro-scale while economic or physical access, dietary diversity (utilization), and coping strategies (stability) are typically measured on micro-scales [5,70]. Every indicator yields important insights, but sizeable gaps remain between the multifaceted nature of food security and what existing measurements reveal about the concept [39,41]. As the concept of food security has begun to be expanded to include sustainability and agency, questions about measurements of these indicators have been brought up [37].

As no single measure is adequately represents all aspects of food insecurity, many organizations have begun triangulating their use of measurements and indicators [68,71]. Some have found value in utilizing indirect measurements such as food balance sheets, temperature, rainfall, marketing data, political instability, trade policies, or household size and dependency ratios to capture the concept more fully [1,5,39]. Unfortunately, some have erroneously used measurements interchangeably, failing to recognize that not all methods capture the same thing [5,39]. To tackle this issue, some have recommended that interchangeable measures of food security be developed [71].

### 2.5. Common Measures of Food Insecurity

In the ensuing sections, we present a few of the most common measurements of food security in the United States with their associated advantages and limitations to highlight how difference in measurements may impact our understanding of food security.

#### 2.5.1. US Household Food Security Survey Module and Food Insecurity Experience Scale

The US Household Food Security Survey Module (HFSSM) and Food Insecurity Experience Scale (FIES) are two of the most used measures of food insecurity in the United States. They are similar experience-based metrics that directly ask people about their access to adequate food in a given reference period [68]. Both measures use yes-no questions to determine individual’s level of food insecurity, starting with the least severe consequences of food insecurity (worry about acquiring food) and progressively moving towards more severe consequences (hunger). To illustrate, the first question of the FIES asks “In the last year was there a time that you were worried you would not have enough food to eat because of a lack of money or other resources?”, the third question asks, “In the last year was there a time when you only ate a few kinds of foods because of a lack of money or other resources?” the fifth question asks “In the last year was there a time when you ate less than you thought you should because of a lack of money or other resources?”, and the eighth and final question of the FIES asks, “In the last year was there a time when you went a whole day without eating because of a lack of money or other resources?” [76]. The HFSSM follows a very similar structure, but for households with children, it asks an additional ten questions that specifically relate to children’s access to food [28]. Using data collected by these measures, it is possible to estimate how many people in a given population are experiencing food insecurity, as well as how extreme their experience with food insecurity may be [68,77,78]. 

While similar in many ways, the HFSSM and the FIES do have some differences. Of the two, the HFSSM (also known as the Core Food Security Module (CFSM)) was developed first by the US Department of Agriculture through the Community Childhood Hunger Identification Project in 1995 [49,68]. Since that time, the HFSSM has been widely used to research the causes and consequences of food insecurity at the household level in the United States and is administered today as part of the monthly Current Population Survey (CPS) and the bi-annual National Health and Nutrition Examination (NHANES) [20,69]. As was mentioned, the HFSSM can be adapted for families with (18 questions) and without children (8 questions). Its reference period can also be adapted, although typically it is used to gather data about people’s experience with food insecurity in the past year or in the past 30 days [49]. It (along with a shortened 6-item version) has been validated in effectively measuring people’s psychological experience with and economic access to food but falls short in adequately capturing other dimensions of food access and other pillars of food security [38,47,79]. The HFSSM is also lengthy and cannot be used to measure food security on an individual level; therefore, it fails to provide an effective cross-national measure of food insecurity. 

The FIES was developed by the Food and Agriculture Organization (FAO) through the Voices of the Hungry Project in 2014 [80]. Its similarity to the HFSSM can be attributed to its basis in both the HFSSM and Latin America and Caribbean Food Security Scale (ELCSA) [68]. In fact, the FIES was specifically developed to address some of the shortfalls of the HFSSM. Accordingly, the FIES is a shorter, more standardized experience-based measure of food insecurity that can be adapted to assess food insecurity at either the individual or household level. Going one step further than the HFSSM, it can be used to measure the percentage of individuals in the population who have experienced food insecurity at moderate or severe levels during a given reference period [80,81]. Initially administered in the Gallup Poll, the FIES has been recognized as a valuable global measure and has been translated into 170 languages and dialects. It has been culturally attuned and provides a standardized measurement of food security across countries despite vast cultural and linguistic differences [76,80]. As the FIES has become increasingly accepted worldwide, it has been used by the FAO (in conjunction with the PoU) to measure the world’s progress toward its Sustainable Development Goals [68,80]. Notable strengths and weaknesses of the FIES are listed below (see Table 4).

#### 2.5.2. Household Food Insecurity Access Scale and Household Hunger Scale

Another common measure is the Household Food Insecurity Access Scale (HFIAS). The HFIAS was developed by the United States Agency for International Development (USAID) and is like the HFSSM and FEIS in that it is primarily used to measure a household’s access to food [68,71]. It has a 30-day recall period and includes nine questions about psychological and behavioral factors influencing food security at the household level [49,70]. These questions ask about people’s feelings of uncertainty or anxiety related to food supply, as well as the preferability, variety, and quantity of foods consumed at the household level [82]. While the HFIAS was initially developed for program monitoring and impact evaluation, it has been used in other circumstances as well [49]. Critics of the HFIAS have pointed out that the reports gathered through this method are subjective and not necessarily applicable across cultures [5]. 

In response to these critiques, an improved version of the HFIAS, the Household Hunger Scale (HHS), was recently developed. The HHS is very similar to the HFIAS, but only has three questions that measure the most severe aspects of food insecurity [5]. As more severe experiences of food insecurity are less subjective (e.g., running out of food is less subjective than feeling anxious about running out of food), the HHS is believed to provide more comparable results across countries and contexts [49]. Despite this improvement, both the HFIAS and HHS have additional weaknesses, the most notable being that they both fail to adequately measure other pillars of food security such as availability, utilization, or stability.

#### 2.5.3. Prevalence of Undernourishment

Food availability is often assessed using the Prevalence of Undernourishment (PoU) indicator [49]. The PoU measure utilizes national food supply, consumption, and energy needs data to estimate the percentage of a country’s population whose typical food intake is below minimal consumption levels [5,68,72]. The FAO uses this indicator (in conjunction with the FIES) to estimate the number of people who are likely not eating enough food to meet their dietary energy needs at the national level [68,78]. This indicator is useful in identifying countries in need and in making comparisons at the national level, but it does not adequately identify vulnerable populations as it provides no specific data on what households or individuals are food insecure or where those experiencing it live [5,29,68,78].

#### 2.5.4. Coping Strategy Index

The Coping Strategy Index (CSI) was developed by the World Food Program and the Cooperative for Assistance and Relief Everyone International (CARE International) and is used to assess the frequency and severity of coping behaviors taken in response to the experience of food insecurity [5,39,70]. It is used by a variety of organizations to help identify at-risk and food insecure households [49,70]. No “universal CSI” exists as every nation, region, and locality has different cultural norms and coping strategies that are engaged in when responding to the experience of food insecurity [5,49]. Accordingly, various culturally attuned versions of the CSI have been developed and are used in different areas of the world [5]. For instance, in more developed nations, a Reduced Coping Strategy Index (rCSI) is often used. This rCSI only measures a couple common, but less-severe coping behaviors, in contrast to the CSI which includes measures of more severe coping behaviors [70]. Because of the variations that exist between CSI measurement tools, it is only to be used as a comparative indicator within a specified geographic area [5]. While the CSI helps policymakers understand people’s behavioral responses to food insecurity in a given region, it does not fully capture other important aspects of food security [49].

#### 2.5.5. Household Dietary Diversity Score and Food Consumption Score

The Household Dietary Diversity Score (HDDS) and Food Consumption Score (FCS) are used to assess food utilization. Both measures analyze the quantity and diversity of foods consumed within a given reference period [70]. The HDDS was developed as part of the Food and Nutrition Technical Assistance Project and assesses the number of food groups that a household has consumed in the last 24 h [5]. It can be adapted to specifically capture the experience of individuals (IDDS) or women (WDDS) and is predominately used by the FAO and USAID [70]. Notably, the HDDS has no predetermined cut-off point in establishing an adequate level of dietary diversity. 

Like the HDDS, the FCS also assesses the number of food groups that a household has consumed, but typically has a seven-day reference period. The FCS also measures the quantity of foods consumed, applying weights to different food groups relative to their nutritional value [70]. The FCS does have a predetermined cut off point in determining who is or is not consuming adequate levels of dietary diversity, but these cutoffs are relatively arbitrary [49]. The FCS is most often distributed by the World Food Programme [70]. While extremely valuable, the FCS only captures dietary diversity on the household level and does not account for differences in the intrahousehold consumption of nutrients [70].

## 3. Limitations

### Measurement Limitations

Despite the progress that has been made in recent years in measuring food security, significant limitations still exist in assessing these concepts. Most measures require significant time, resources, and technological expertise to be understood and analyzed [68]. In addition to the unavoidable complications accompanying any study involving human participants, a researcher’s insufficient understanding of an indicator or combination of indicators may result in a blindly inaccurate representation of food security. Even with a perfect understanding of indicators, however, measurement specific shortcomings complicate the process of determining people’s food security status [5,83]. 

Many conventional efforts used to gather data using these indicators overlook the experiences of ‘essential workers,’ homeless individuals, migrants, refugees, or others living in marginal housing or on reserves [4,12,20,29,61,84]. This under coverage bias is concerning as these groups are often more likely to report experiencing food insecurity [18]. The length of many food security measurements can also affect the representativeness of samples. While answering the HFSSM’s 18 questions has been found to only take an average of four minutes, being presented with such a lengthy questionnaire can still be overwhelming [34]. Partially because of this, in times of recent crisis, the USDA has chosen to rely upon a one-item food sufficiency question in the Household Pulse Survey (HPS) to roughly estimate household’s access to food [28,85].

## 4. Conclusions

Although valiant efforts are underway to combat world hunger and reduce the number of malnourished people, further progress will depend on clarifying the semantics of food security and food insecurity. Despite the ratification of universal definitions in the 1990s, which were meant to curb the development of new definitions, the terms have continued to diverge due to the multifaceted nature of the concepts [86,87]. While most definitions of food security/insecurity used today highlight similar points, slight, yet impactful, semantic differences remain, hindering researchers, practitioners, and policymakers from effectively measuring and finding solutions to food insecurity. By recognizing such semantic differences and the corresponding challenges of existing measurements of food insecurity, policy makers and practitioners can create effective policies that will reverse the rising rates of food insecurity the world is seeing today. Without re-emphasizing the use of consistent definitions and measures of food insecurity, effective actions and policies to combat hunger will remain beyond our reach.

## Figures and Tables

**Table 1:** Levels of analysis.

Level of Analysis	Description
Global	Refers to the availability and reliability of food worldwide.
National	Refers to the availability and reliability of food at a national level, the production of food within countries, and the levels of food reserves that should be maintained consistently.
Regional	Refers to the availability and reliability of food at a regional level.
Household	Refers to a household’s physical and economic access to food, their levels of vulnerability, and their utilization of food.
Individual	Refers to an individual’s physical and economic access to food (recognizing that food is not always evenly distributed at the household level), their levels of vulnerability, and their utilization of food.

Note: The table gives descriptions of different levels in which experiencing hunger is measured.

**Table 2:** Different Interpretations of ‘Enough’.

Citation	Definition
Minimal levels of food consumption	
Alamgir and Arora 1991 [51]	The sum of household and sub-national food security, and more. At the national level, food security can be defined as assured national availability of food to meet current *minimum requirements per capita* during a reference period (normally, one year) and, also, to meet any unexpected shortfall over a limited period (e.g., three months).
IFAD, 1991 [52]	Access to enough food to ensure the *minimum necessary food intake* for all individual members to lead a healthy life.
Siamwalla and Valdes 1980 [53]	The ability to meet *target levels of consumption* on a yearly basis.
World Bank Staff, 1980 [54]	The assurance of a *minimally adequate level of food consumption*.
**Necessary to meet nutritional needs**	
Alaimo, 2005 [18]	Food insecurity: limited or uncertain availability of *nutritionally adequate* or safe foods.
Barraclough and Utting 1987 [55]	Assured access by all social groups and individuals to food *adequate in quality and quantity to meet nutritional needs*.
Benson et al., 1986 [56]	Having assured sets of entitlements—from food production, cash income, reserves of food or assets, and/or government assistance programs—such that in times of need people will be able to maintain *sufficient nutritional intake for physical well-being*.
Eicher and Staatz, 1986 [57]	The ability of a country or region to assure, on a long-term basis, that its food system provides the total population access to a timely, reliable, and *nutritionally adequate supply of food*.
Jonsson and Toole, 1991 [58]	Access to food, *adequate in quantity and quality, to fulfill all nutritional requirements* for all household members throughout the year.
Life Sciences Research Office and Andersen, 1990 [24]	Food insecurity: the limited or uncertain availability of *nutritionally adequate* and safe foods or limited or uncertain ability to acquire acceptable foods in socially acceptable ways.
Winne et al., 2000 [59]	All persons in a community have access to culturally acceptable, *nutritionally adequate* food through local non-emergency sources at all times.
**The amount of food needed for an “active, healthy life”**	
Bartfeld and Dunifon, 2006 [60]	The assured access of all people to *enough food for a healthy and active life.*
Coleman-Jensen et al., 2021 [61]	Consistent, dependable access to *enough food for active, healthy living*.
FAO et al., 2009 [2]	Food security exists when all people, at all times, have physical, social, and economic access to sufficient, safe, and nutritious food that meets their dietary needs and food preferences *for an active and healthy life*.
Haddad et al., 1995 [62]	Availability of sufficient food at all times for all people to *ensure an active and healthy life.*
Hayes, 2021 [26]	Consistent access to *enough food for active, healthy lives* for all household members at all times during the year.
Kabeer, 1990 [63]	The ability of a household to assure all its members sustained access to *sufficient quantity and quality of food to live active, healthy lives*.
Life Sciences Research Office and Andersen, 1990 [24]	Access by all members at all times to *enough food for an active, healthy life.* Food security includes at a minimum:the ready availability of nutritionally adequate and safe foods; assured ability to acquire acceptable foods in socially acceptable ways (i.e., without resorting to emergency food supplies, scavenging, stealing, or other coping strategies).
Pinstrup-Andersen, 2009 [50]	Access by all people to *enough food to live a healthy and productive life*.
Reutlinger, 1985 [64]	Access by all people at all times to *enough food for an active and healthy life.*
Reutlinger, 1986 [65]	Access by all people at all times to *enough food for an active, healthy life*.
United Nations, 1990 [66]	The ability of household members to assure themselves sustained access to a *sufficient quantity and quality of food to live active, healthy lives*.
World Food Program, 1996 (2009) [67]	When all people, at all times, have physical and economic access to sufficient, safe and nutritious food that meets their dietary needs and food preferences *for an active and healthy life*.

Note: The table gives definitions and citations of different interpretations of ‘enough’ in the literature.

**Table 3:** Measures of food security in the United States (2022).

Measure	Description
Anthropometric measures	Measures bodily characteristics such as height, weight, skinfold, etc. (combined with weight and age) to assess the utilization of food [5]
Coping Strategies Index (CSI)	Measures the frequency and severity of context specific behaviors taken in response to food insecurity [5]
Domestic Food Price Volatility	Measures the variability in the annual food price index [5]
Food Consumption Score (FCS)	Measures the quality, quantity, and diversity of foods consumed by a household in the last seven days [70]
Food Insecurity Experience Scale (FIES)	Measures the severity of food insecurity in population groups based off individual or household level data [68]
Global Food Security Index (GFSI)	Measures the affordability, availability, quality, resiliency, and safety of available foods in given nations [15]
Global Hunger Index (GHI)	Measures rates of undernourishment, underweight children, and child mortality at the national level to assess hunger [68,70]
Household Consumption and Expenditure Surveys (HCES)	Measures a variety of household socioeconomic conditions affecting food security such as food acquisition and consumption [5]
Household Dietary Diversity Scores (HDDS)	Measures the number of food groups (grains, starches, vegetables, fruits, meat, eggs, fish, legumes, dairy, fats, sugar, condiments, etc.) consumed by an individual or household in the previous 24 h [71]
Household Food Insecurity Access Scale (HFIAS)	Measures the frequency and intensity of challenges that households face in accessing food [71]
Household Food Security Survey Module (HFSSM)	Measures the prevalence of food insecurity among households in the United States [49]
Household Pulse Survey (HPS)	Measures household sufficiency of food in the last 7 days, 2 weeks, or month based off a single question [26,28]
Months of Adequate Food Provisioning (MAHFP)	Measures a household’s stability in maintaining adequate levels of food in the past year [5,71]
Prevalence of Undernourished (PoU)	Measures the percentage of a country’s population whose typical food intake is below the levels needed for an active and healthy life using national estimates [68,72]
Relative Dietary Supply Index	Compares the available dietary energy supply in a country with that country’s average caloric needs [5]
Sustainable Nutrition Security (SNS)	Incorporates metrics of sustainability [73]
Women’s Empowerment Nutrition Grid (WEN or WENI)	Incorporate agency, alongside knowledge and resources, as dimensions of empowerment with respect to food security [37,74,75]

Note: The table gives measures and their descriptions of food security in the United States.

**Table 4:** Strengths and weaknesses of the FIES.

Strengths	Weaknesses
Helps identify risk factors and consequences of food insecurity. Can assist in identifying vulnerable populations before times of crisis. Effectively captures psychosocial aspects of food security. Simple, time effective, and relatively inexpensive Provides a standardized and comparable global measurement. Allows disaggregation of data by gender. Can be used in combination with other indicators. Has been effectively translated into many languages.	Does not capture the full range of food security. Does not measure diet quality, food consumption, or expenditures. May be challenging for non-specialists to analyze data. Assumes that the process of food insecurity is orderly and predictable across all cultures. Is relatively new.

Note: Author’s compilation. Source: [68,72,80].

## Data Availability

No new data were created or analyzed in this study. Data sharing is not applicable to this article.

## Appendix

The Table A1 and Table A2 provide definitions of food security present in the literature since 1996 and 2000, respectively. The tables provide the citation, level of analysis (individual, national, regional, etc.), and definition.

The Table A3, Table A4 and Table A5 provide definitions and their citation of different ideas of ‘adequate food’, ‘preferred foods’ and ‘culturally or socially acceptable means’ based on the literature, respectively.

**Table A1:** Definitions of Food Security Used in the Literature Since 1996.

Citation	Level of Analysis	Definition
Bartfeld and Dunifon, 2006, p. 921 [60]	Individual	Assured access of all people to enough food for a healthy and active life.
Béné, 2020 [88]	Individual, Household	When individuals and households have adequate resources to obtain appropriate food
Blumberg et al., 1999, p. 1231 [79]	Individual	Assured access to nutritionally adequate and safe foods without resorting to emergency food supplies, scavenging, stealing, and other coping strategies.
Coleman-Jensen et al., 2021 [61]	Individual	Consistent, dependable access to enough food for active, healthy living.
Committee on World Food Security, 2012 [46]	Individual	When all people at all times have physical, social, and economic access to food, which is safe and consumed in sufficient quantity and quality to meet their dietary needs and food preferences, and is supported by an environment of adequate sanitation, health services and care, allowing for a healthy and active life.
Wood et al., 2000 [89]	Individual	The state in which all persons obtain a nutritionally adequate, culturally acceptable diet at all times through local, non-emergency sources.
FAO et al., 2009 [2]	Individual	Food security exists when all people, at all times, have physical, social, and economic access to sufficient, safe, and nutritious food that meets their dietary needs and food preferences for an active and healthy life.
Hayes, 2021 [26]	Individual	Consistent access to enough food for active, healthy lives for all household members at all times during the year.
Life Sciences Research Office and Andersen, 1990 [24]	Individual	Access by all members at all times to enough food for an active, healthy life. Food security includes at a minimum: the ready availability of nutritionally adequate and safe foods; assured ability to acquire acceptable foods in socially acceptable ways (i.e., without resorting to emergency food supplies, scavenging, stealing, or other coping strategies).
Pinstrup-Andersen, 2009 [50]	Global, National, Regional, Household	Enough food is available, whether at the global, national, community, or household level.
Pinstrup-Andersen, 2009 [50]	Individual	Access by all people to enough food to live a healthy and productive life.
Prifti, 2021, p. 238 [72]	National, Regional, Household, Individual	A function of availability of adequate food in terms of quantity and quality and the people’s ability to afford it at all times.
Siche, 2020 [90]	Individual	Everyone has unrestricted access to food that allows them to satisfy their basic needs.
Sustainable Development Commission, 2009, p. 10 [91]	National	The aspiration for genuinely sustainable food systems, where the core goal is to feed everyone sustainably, equitably, and healthily; which addresses needs for availability, affordability, and accessibility; which is diverse, ecologically sound, and resilient; and which builds the capabilities and skills necessary for future generations.
Committee on World Food Security, 2012 [46]	Individual	When all people, at all times, have physical, social, and economic access to sufficient, safe, and nutritious food that meets their dietary needs and food preferences for an active and healthy life… The four pillars of food security are availability, access, utilization, and stability. The nutritional dimension is integral to the concept of food security.
Winne et al., 2000, p. 4 [59]	Regional	All persons in a community have access to culturally acceptable, nutritionally adequate food through local non-emergency sources at all times.
World Food Program, 2009, p. 170 [67]	Individual	A condition that exists when all people, at all times, are free from hunger.
World Food Summit, 1996 [2]	Individual	When all people, at all times, have physical and economic access to sufficient, safe, and nutritious food that meets their dietary needs and food preferences for an active and healthy life.

Note: While not all-inclusive, the definitions presented illustrate the need for a comprehensive definition. To our knowledge, all definitions are reported verbatim and are cited with their original source.

**Table A2:** Definitions of Food Insecurity Used in the Literature Since 2000.

Citation	Level of Analysis	Definition
Alaimo, 2005 [18]	Global, National, Regional	Limited or uncertain availability of nutritionally adequate or safe foods.
Babu and Gajanan, 2021 [31]	Individual	A lack of access to the kinds and amounts of food necessary for each member of a household to lead an active and a healthy lifestyle.
Bergmans, 2019 [92]	Individual	The physical pain of hunger as well as the more common experience of worrying about having enough healthy food to eat.
Bovell et al., 2015 [93]	Household	Limited or uncertain access to enough food for all household members to live active and healthy lives.
Coleman-Jensen et al., 2014 [94]	Household	A household-level economic and social condition of limited or uncertain access to adequate food.
Coleman-Jensen et al., 2021 [61]	Individual	Access to adequate food is limited by a lack of money and other resources.
Davitt et al., 2021 [95]	Household	Diminished variety, quality, and desirability of diet as well as decreased access to food.
Donley and Gualtieri, 2015 [96]	Individual	Lacking enough money to buy the amount and variety of food one needs or wants.
Dowler et al., 2001 [97]	Individual	The inability to acquire or consume an adequate quality or sufficient quantity of food in socially acceptable ways, or the uncertainty that one will be able to do so.
Economic Research Service, 2022 [28]	Household	Households were, at times, unable to acquire adequate food for one or more household members because the household had insufficient money and other resources for food.
FAO et al. 2022 [3]	Individual	Lack regular access to enough safe and nutritious food for normal growth and development and an active and healthy life. This may be due to unavailability of food and/or lack of resources to obtain food.
Food Forward, 2019 [98]	Household, Individual	Lack of access to enough, good, healthy, and culturally appropriate food.
Frongillo and Horan, 2004, p. 28 [47]	Global, National, Regional, Household, Individual	Problems with the availability, accessibility, and utilization of food.
Gundersen and Ziliak, 2015, p. 1830 [13]	Household	A condition which households lack access to adequate food because of limited money or other resources.
Harke et al., 2021, p. 1 [99]	Household, Individual	The lack of access to sufficient food because of limited financial resources.
Life Sciences Research Office and Andersen, 1990 [24]	National, Regional, Household	The limited or uncertain availability of nutritionally adequate and safe foods, or limited or uncertain ability to acquire acceptable foods in socially acceptable ways.
Litton and Beavers, 2021 [100]	Individual	Reduced quality, variety, or desirability of diet.
Mardones et al., 2020 [101]	Individual	Unreliable physical, social, and economic access to sources of adequate and nutritious food that meets people’s dietary needs and food preferences.
Maxwell et al., 1990 [102]	Individual	The lack of access to enough food.
Miriam Webster Dictionary, 2022 [30]	Household, Individual	The fact or an instance of being unable to consistently access or afford adequate food.
National Research Council et al., 2006 [8]	Household, Individual	Individuals and/or families in a household adjusting their dietary intakes or preferences because of a lack of physical or economic resources.
National Research Council, 2006, p. 4 [8]	Household, Individual	Uncertain, insufficient, or unacceptable availability, access, or utilization of food.
National Research Council, 2006, p. 44 [8]	Individual	The social and economic problem of lack of food due to resource or other constraints, not voluntary fasting, or dieting, or because of illness, or for other reasons.
Niles et al., 2020 [103]	Household, Individual	The lack of consistent physical, social, and economic access to adequate and nutritious food that meets dietary needs and food preferences.
Nord and Prell, 2007 [104]	Household	Household level economic and social condition of limited access to food.
Nord et al., 2005 [105]	Household, Individual	The disruption of food intake or eating patterns because of lack of money and other resources.
Power et al., 2016, p. 4 [106]	Household, Individual	Inadequate or insecure access to adequate food due to financial constraints.
Roshanafshar and Hawkins, 2015, p. 4 [107]	Household	When one or more (household) members do(es) not have access to the variety or quantity of food that they need due to lack of money.
Shapouri, 2010, p. v [108]	Individual	Consuming less than the nutritional target of 2100 calories per day per person.
United Health Foundation, 2022 [17]	Household	Households unable to provide adequate food for one or more household members due to lack of resources.
US Department of Agriculture, 2022 [27]	Household	Lack of available financial resources for food at the household level.
US Department of Agriculture, 2022 [27]	Household	A household’s inability to provide enough food for each person to live an active healthy lifestyle.
Voices for Alabama’s Children, 2015 [109]	Household	Lack of access, at times, to enough food for an active, healthy life for all household members and limited or uncertain availability of nutritionally adequate foods.

Note: While not all-inclusive, the definitions presented illustrate the need for a comprehensive definition. To our knowledge, all definitions are reported verbatim and are cited with their original source.

**Table A3:** Different Ideas of ‘Adequate Food’.

Citation	Definition
Dietary Needs	
Shapouri, 2010, p. v [108]	Consuming less than the nutritional target of *2100 calories per day* per person.
**Nutritious (and Safe)**	
Alaimo, 2005 [18]	Food insecurity: limited or uncertain availability of *nutritionally adequate* or *safe foods.*
Life Sciences Research Office and Andersen, 1990 [24]	Food insecurity: whenever the availability of *nutritionally adequate and safe foods* or the ability to acquire acceptable foods in socially acceptable ways is limited or uncertain.
Blumberg et al., 1999, p. 1231 [79]	Assured access to *nutritionally adequate* and *safe* foods without resorting to emergency food supplies, scavenging, stealing, and other coping strategies.
Wood et al., 2000 [88]	The state in which all persons obtain a *nutritionally adequate*, culturally acceptable diet at all times through local, non-emergency sources.
Life Sciences Research Office and Andersen, 1990 [24]	Access by all members at all times to enough food for an active, healthy life. Food security includes at a minimum: the ready availability of *nutritionally adequate* and *safe* foods; assured ability to acquire acceptable foods in socially acceptable ways (i.e., without resorting to emergency food supplies, scavenging, stealing, or other coping strategies).
**Quality**	
Prifti, 2021, p. 238 [72]	A function of availability of adequate food in terms of quantity and *quality* and the people’s ability to afford it at all times.
**Multifaceted**	
Winne et al., 2000, p. 4 [59]	All persons in a community have access to *culturally acceptable, nutritionally adequate* food through local non-emergency sources at all times.
Committee on World Food Security, 2012 [46]	When all people at all times have physical, social, and economic access to food, which is *safe* and consumed in sufficient quantity and *quality* to meet their *dietary needs* and *food preferences*, and is supported by an environment of adequate sanitation, health services and care, allowing for a healthy and active life.
Davitt et al., 2021 [94]	Food insecurity: diminished *variety, quality, and desirability* of diet as well as decreased access to food.
Donley and Gualtieri, 2015 [95]	Food insecurity: lacking enough money to buy the amount and *variety* of food one *needs* or *wants.*
FAO, 2009 [2]	Food security exists when all people, at all times, have physical, social, and economic access to *sufficient, safe,* and *nutritious* food that meets their *dietary needs* and *food preferences* for an active and healthy life.
Food Forward, 2019 [97]	Food insecurity: lack of access to enough, *good*, healthy, and *culturally appropriate food.*
Niles et al., 2020 [103]	Food insecurity: the lack of consistent physical, social, and economic access to adequate and *nutritious* food that meets *dietary needs* and *food preferences.*
World Food Summit, 1996 [2]	When all people, at all times, have physical, social, and economic access to *sufficient*, *safe*, and *nutritious* food that meets their *dietary needs* and *food preferences* for an active and healthy life.

**Table A4:** Different Ideas of ‘Preferred Foods’.

Citation	Definition
Tastes and Preferences	
Mardones et al., 2020 [101]	Food insecurity: unreliable physical, social, and economic access to sources of adequate and nutritious food that meets people’s dietary needs and *food preferences.*
Niles et al., 2020 [103]	The lack of consistent physical, social, and economic access to adequate and nutritious food that meets dietary needs and *food preferences.*
**Desirability**	
Davitt et al., 2021 [95]	Food insecurity: diminished variety, quality, and *desirability of diet* as well as decreased access to food
Donley and Gualtieri, 2015 [96]	Food insecurity: lacking enough money to buy the amount and variety of food one needs or *wants.*
Litton and Beavers, 2021 [100]	Food insecurity: reduced quality, variety, or *desirability of diet.*
**Culturally Acceptable**	
Winne et al., 2000, p. 4 [59]	All persons in a community have access to *culturally acceptable*, nutritionally adequate food through local non-emergency sources at all times.
Wood et al., 2000 [89]	The state in which all persons obtain a nutritionally adequate, *culturally acceptable diet* at all times through local, non-emergency sources.
Food Forward, 2019 [98]	Food insecurity: lack of access to enough, *good*, healthy, and *culturally appropriate food.*

**Table A5:** Different Ideas of ‘Culturally or Socially Acceptable Means’.

Citation	Definition
Non-Emergency Means	
Winne et al., 2000, p. 4 [59]	All persons in a community have access to culturally acceptable, nutritionally adequate food through local *non-emergency sources* at all times.
Blumberg et al., 1999, p. 1231 [79]	Assured access to nutritionally adequate and safe foods *without resorting to emergency food supplies, scavenging, stealing, and other coping strategies.*
Wood et al., 2000 [89]	The state in which all persons obtain a nutritionally adequate, culturally acceptable diet at all times through *local, non-emergency sources.*
Life Sciences Research Office and Andersen, 1990 [24]	Access by all members at all times to enough food for an active, healthy life. Food security includes at a minimum:the ready availability of nutritionally adequate and safe foods; assured ability to acquire acceptable foods in socially acceptable ways (i.e., *without resorting to emergency food supplies, scavenging, stealing, or other coping strategies*).
**Monetary Means**	
United Health Foundation, 2022 [17]	Food insecurity: households unable to provide adequate food for one or more household members due to *lack of resources,*
Coleman-Jensen et al., 2021 [61]	Access to adequate food is limited by a *lack of money and other resources.*
Donley and Gualtieri, 2015 [96]	Food insecurity: lacking enough *money to buy* the amount and variety of food one needs or wants.
Economic Research Service, 2022 [28]	Food insecurity: household members were, at all times, unable to acquire adequate food for one or more household members because the household had *insufficient money and other resources for food.*
Harke et al., 2021, p. 1 [99]	The lack of access to sufficient food because of *limited financial resources.*
Gundersen and Ziliak, 2015, p. 1830 [13]	Food insecurity: a condition which households lack access to adequate food because of *limited money or other resources.*
Office of Disease Prevention and Health Promotion, 2020 [110]	Food insecurity: the disruption of food intake or eating patterns because of *lack of money and other resources.*
USDA, 2021 [111]	Food insecurity: lack of available *financial resources for food* at the household level.
